# Taraxasterol ameliorates dextran sodium sulfate-induced murine colitis via improving intestinal barrier and modulating gut microbiota dysbiosis: Taraxasterol ameliorates colitis via intestinal barrier and gut microbiota dysbiosis

**DOI:** 10.3724/abbs.2022019

**Published:** 2022-03-03

**Authors:** Chen Li, Meng Wang, Xiqi Chen, Wei Chen

**Affiliations:** 1 Department of General Surgery Affiliated Hospital of Shandong University of Traditional Chinese Medicine Jinan 250014 China; 2 Department of Gastroenterology Shuguang Hospital Affiliated to Shanghai University of Traditional Chinese Medicine Shanghai 201203 China

**Keywords:** taraxasterol, colitis, intestinal barrier, 16S rDNA sequencing, gut microbiota

## Abstract

Taraxasterol (TAX) has been proven to prevent and treat inflammatory diseases. However, the effects of TAX on intestinal barrier and the diversity, structure, and function of gut microbiota have yet to be elucidated in dextran sodium sulfate (DSS)-induced colitis mice. Our objectives are to evaluate the effect of TAX on intestinal barrier and its impact on gut microbiota. Herein, immunofluorescence analysis is conducted to determine the expressions of tight junction (ZO-1) and mucin (Mucin-2) proteins. The abundance, diversity, and function of fecal colonies are investigated by using 16S rDNA sequencing, and the influence of TAX on the gut microbiota in mice is also analyzed. Our results suggest that TAX attenuates the symptoms in DSS-induced colitis mice by reducing the DAI score, increasing colon length, alleviating histopathological damage of colon tissues, and improving intestinal barrier. 16S rDNA sequencing of fecal samples indicates that TAX intervention has a regulatory effect on DSS-induced gut microbiota dysbiosis at different taxonomic levels. TAX increases microbial diversity that is reduced by DSS. It normalizes the relative abundance of *Bacteroidetes* and the ratio of *Bacteroidetes*/ *Firmicutes*. In addition, treatment with TAX has a better effect on the function of metabolisms, such as nucleotide, lipid, and bile acid metabolism. These findings suggest that TAX may be a good candidate for the remission of colitis, which is related to improving intestinal barrier and modulating gut microbiota.

## Introduction

Ulcerative colitis (UC), one form of inflammatory bowel disease (IBD), is an inflammatory destructive disease characterized by abdominal pain, severe diarrhea, and rectal bleeding [1]. Inflammation usually occurs in the rectum and lower part of the colon, even affecting the entire colon. The incidence and prevalence of UC are increasing worldwide, and it has evolved into a global burden [2]. Patients with UC suffer from a worse quality of life and a higher risk of colorectal cancer than the general population [2]. Hence, it is necessary to explore the underlying pathogenesis of UC and develop effective therapy.

The etiology of UC is complex and involves various factors such as genetics, environment, epithelial barrier defects, immune system disorders, and gut microbiota [3]. Especially, abundant evidence suggests that elevated dysfunctions of the intestinal barrier and gut microbiota are central events in the pathogenesis of UC [ 4, 5]. The current treatment paradigm for UC is to achieve clinical remission and mucosal healing and involves the use of 5-aminosalicylic acid, immunomodulators, and other biological agents [2]. Although a number of drugs are available, not all patients achieve sustained remission, and adverse reactions caused by drugs usually limit the use of drugs [6]. Therefore, it is necessary to explore therapeutic drugs with efficacy and safety.

**Figure 1:**
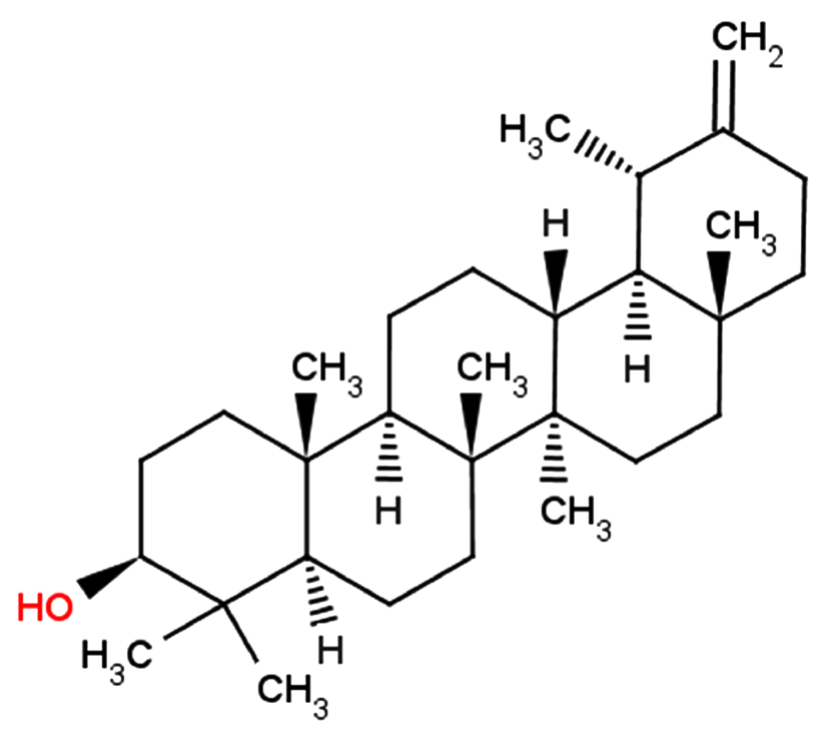
Figure1 The chemical structure of TAX

Taraxacum officinale, one of the most widely used herbal medicines, has been shown to have diverse pharmacological benefits, such as anti-inflammation [7], antioxidant activities [8], and regulating lipid metabolism [9]. Taraxasterol (TAX; Figure 1), the main constituent of *Taraxacum officinale*, is an active compound of pentacyclic-triterpene. In the past few decades, increasing evidence indicated that TAX displays a variety of biological activities in different disease models, including acute kidney injury [10], colorectal cancer [11], and liver injury [12]. Our previous study showed that TAX administration repressed xenograft tumor growth [13] and reduced intestinal inflammation in mice with dextran sodium sulfate (DSS)-induced colitis [14]. However, the role of TAX on intestinal barrier function and gut microbiota of colitis has not been fully studied.

In the present study, we aimed to determine the effects of TAX on intestinal integrity and microbial dysbiosis in DSS-induced colitis. In addition, the related functional pathways of gut microbiota were also investigated. We found that TAX improves intestinal barrier function and regulates the composition and function of gut microbiota in DSS-induced colitis mice.

## Materials and Methods

### Reagents and antibodies

Taraxasterol (purity: 98%) was purchased from Chengdu Preferred Biotechnology (Chengdu, China). DSS (MW 36,000~50,000) was obtained from MP Biomedicals (160110; Santa Ana, USA). The antibodies included anti-ZO-1 antibody (GB111402; Serbicebio, Wuhan, China) and anti-mucin-2 antibody (GB11344; Serbicebio). Carboxymethyl cellulose sodium (CMC−Na) was provided by Sinopharm Chemical Reagent Co. (Shanghai, China).

### Animals

Male C57BL/6J mice (6–8 weeks old, 18–20 g) were purchased from Shanghai Lingchang Biotechnology (Shanghai, China). All mice were given standard pellet feed and water under stable temperature (22±2°C) and relative humidity (45%–50%), and were fed adaptively for 1 week before the experiment. All of the animal experiments were in strict accordance with protocols of the Ethics Committee of the Affiliated Hospital of Shandong University of Traditional Chinese Medicine.

The doses of TAX used in this study were based on our previous study [14] and other reports [ 10, 15]. Different doses of TAX were dispersed in 0.5% CMC−Na. To induce colitis in mice, the mice in the DSS+water, 10 mg/kg TAX, and 5 mg/kg TAX groups were administered with 3.0% ( *w*/ *v*) DSS in drinking water for 7 days. The mice in the control group received drinking water only. At the same time, the mice in the 10 mg/kg TAX and 5 mg/kg TAX groups were administered with the corresponding dose of TAX in 0.2 mL of 0.5% CMC−Na, respectively. The mice in the control and DSS+water groups were administered with the same volume of 0.5% CMC−Na. During the experiment, mice were weighed at 9 o’clock every morning and the conditions of diarrhea, hematochezia, mobility, and hair were observed.

### Histopathological analysis

Histopathological analysis was carried out according to the procedures reported in previous literatures [16]. Briefly, at the end of treatments, the colon tissues were collected from mice, fixed in 4% paraformaldehyde solution for 48 h, and embedded in paraffin. The tissue sections were deparaffinized, stained with hematoxylin-eosin, and examined by microscopy. Histopathological injury scores, including the severity of inflammation (0, none; 1, mild; 2, moderate; and 3, severe), the degree of involvement (0, none; 1, mucosa; 2, mucosa and submucosa; and 3, transparent), and the degree of epithelial/crypt damage (0, none; 1, basic 1/3; 2, basic 2/3; 3, crypt loss; and 4, crypt and surface epithelial damage) were calculated as previously described [17]. Then each parameter was multiplied by a factor that reflects the percentage of colon involved (1, 0%–25%; 2, 26%–50%; 3, 51%–75%; and 4, 76%–100%), and then add up to get the total point. The maximum score is 40 points.

### Immunofluorescence staining

To evaluate the protein expressions of ZO-1 and Mucin-2 in colon tissues, immunofluorescence staining was performed as previously reported [18]. Briefly, colon tissue slides were deparaffinized, rehydrated, and subjected to antigen retrieval. The slides were blocked with BSA, and subsequently incubated with ZO-1 antibodies (1:600) and mucin-2 antibody (1:200) overnight at 4°C. After wash with PBS, the slides were incubated with Alexa Fluor® 488-conjugated goat anti-rabbit IgG (GB25303, 1:400; Serbicebio) and Cy3-conjugated goat anti-rabbit IgG (GB21303, 1:300; Serbicebio), respectively. The nuclei were stained with DAPI. Finally, fluorescence images were visualized and captured under a fluorescence microscope (Eclipse Ti-SR; Nikon, Tokyo, Japan) at a magnification of 100×.

### Alcian blue/periodic acid Schiff (AB/PAS) staining

Briefly, first, the paraffin sections were dewaxed with xylene and absolute ethanol, and then the AB/PAS solution set (Servicebio) was used to stained: sections were stained with AB-PAS C for 15 min, and rinsed with tap water until colorless. Then the sections were stained with AB-PAS B for 15 min, rinsed with tap water, and rinsed twice with distilled water. AB-PAS A at room temperature was used to stain the sections for 30 min in the dark, and the sections were rinsed for 5 min. After dehydration and sealing, the images were observed under the microscope.

Briefly, the luminal stool in mice colon was collected. Fecal sample (200 mg) was suspended in 0.1 M Tris buffer (pH 7.5) containing 4 M guanidine thiocyanate and 10% N-lauroyl sarcosine. Then, DNA extraction was conducted using the bead-beating method, and DNA quality control was performed using the Qubit fluorometer (Q33216; Thermofisher, Shanghai, China). The v3–v4 regions of the 16S rDNA sequence were amplified by PCR. According to the standard instructions, the PCR fragments were sequenced with the 454 GS-FLX platforms (Roche). The operational taxonomic unit (OTU) was obtained based on 97% similarity. QIIME (Version 1.70, http://qiime.sourceforge.net/) [19] was used to draw flora abundance distribution maps and heatmaps, analyze differences in flora, and perform alpha and beta diversity analysis. Cladogram plot was drawn using the Figure Tree software ( http://tree.bio.ed.ac.uk/software/figtree/) to identify the corresponding biomarkers. The LEfSe tool ( https://huttenhower.sph.harvard.edu/galaxy/) was used to perform linear discriminant analysis (LDA) effect size (LEfSe) on the 16S sequence results. PICRUSt2 software ( https://github.com/picrust/picrust2) was used to predict the function of the microflora.

### Statistical analysis

Statistical analysis was performed using GraphPad Prism 8.0.1. Comparison between two groups was performed by *t*-test, and Multiple comparisons among multiple groups were performed by one-way ANOVA. All data were presented as the mean±SD. *P*<0.05 was considered to be statistically significant.

## Results

### TAX improved the symptoms of DSS-induced colitis in mice

**Figure 2:**
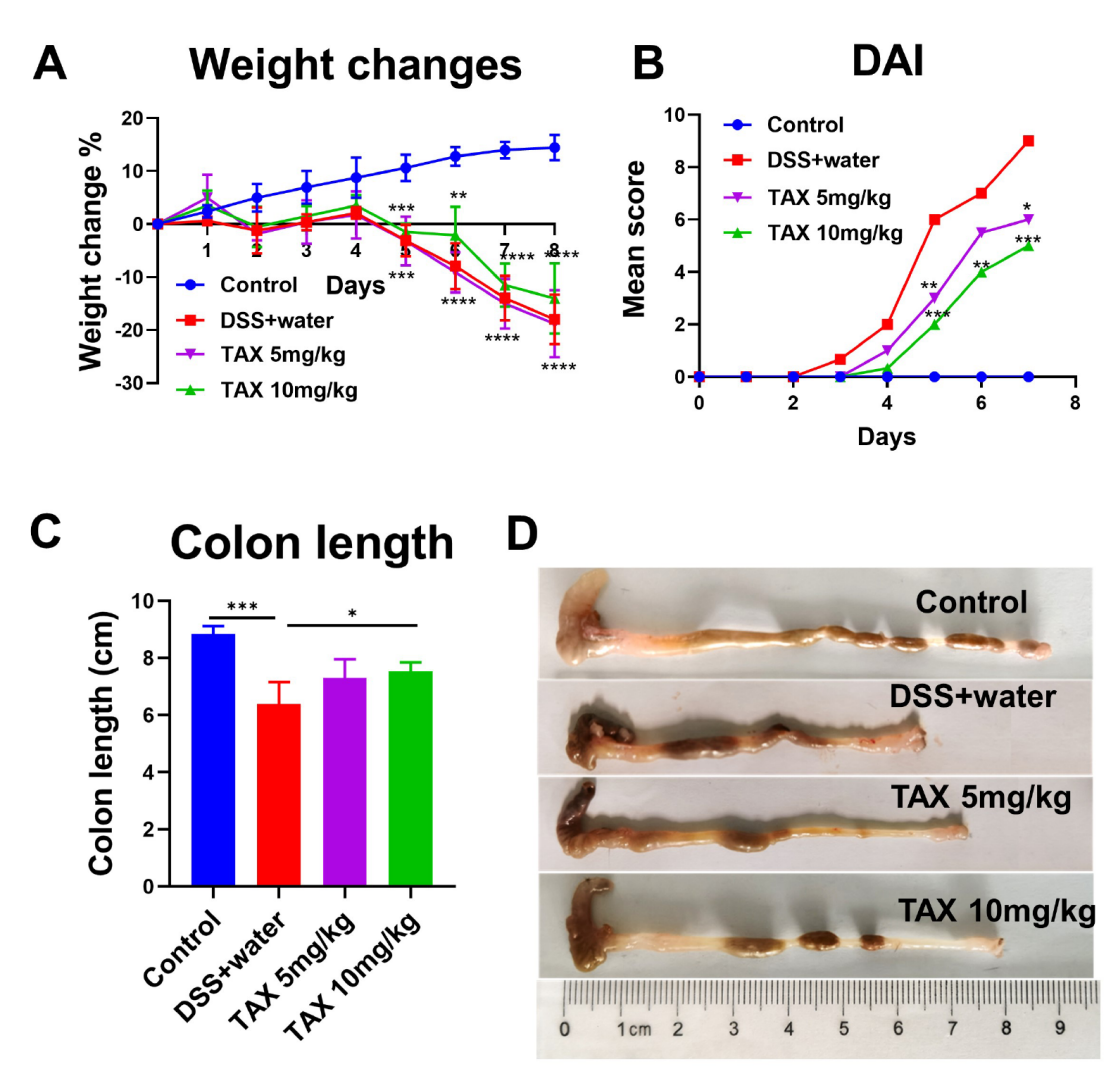
Figure2 TAX attenuated the severity of DSS-induced colitis (A) The weight loss (%). **P<0.01, ***P<0.001, and ****P<0.0001 compared with the control group. (B) DAI score. *P<0.05, **P<0.01, and ***P<0.001 compared with the DSS+water group. (C) Colon length in the four groups. *P<0.05, and ***P<0.001. (D) Representative images of the colon in the four groups. Data are expressed as the mean±SD.

Mice were given a 3% DSS solution for 7 days to establish colitis model and then treated with TAX. The results showed that DSS significantly reduced the body weight of the mice, and 10 mg/kg TAX treatment slowed down the trend of weight loss ( Figure 2A). Compared with that in the control group, the DAI score in the DSS+water group was increased significantly, while treatment with both 10 mg/kg and 5 mg/kg TAX notably inhibited the increase of DAI score ( Figure 2B). Moreover, the colons in the 10 mg/kg TAX group were significantly longer than those in the DSS+water group ( Figure 2C,D). These results indicated that TAX improved the symptoms of DSS-induced colitis in mice.

### TAX attenuated DSS-induced colon injury in mice

**Figure 3:**
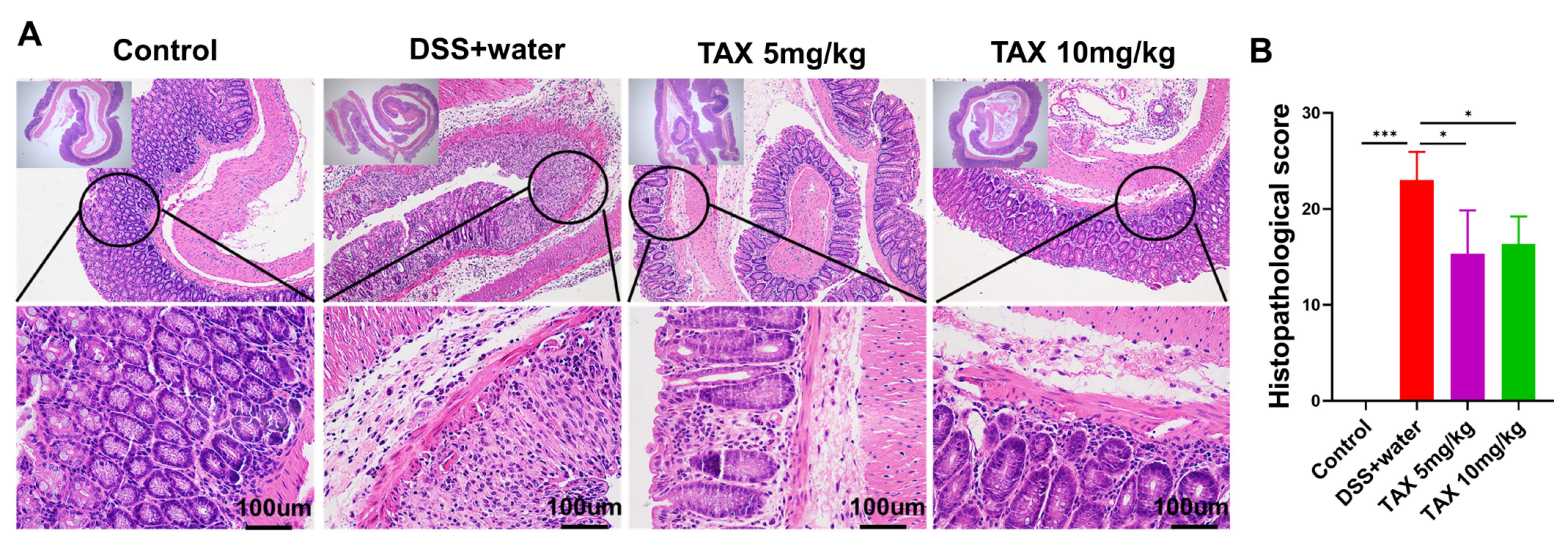
Figure3 TAX alleviated DSS-induced colon histological changes in mice (A) Representative H&E staining images of colon tissue in each group. (B) Histopathological scores were evaluated in each group. Data are expressed as the mean±SD. *P<0.05, ***P<0.001.

To further evaluate the protective effects of TAX on DSS-induced colitis, we performed a histological evaluation of the extent of colonic epithelial damage, submucosal edema, and inflammatory cell infiltration. As shown in Figure 3A, mice developed worsening and extensive colitis after DSS treatment. Oral administration of TAX significantly improved histopathological changes induced by DSS. The histological score further confirmed the protective effect of TAX on colitis mice. Both 10 mg/kg and 5 mg/kg TAX reduced the histopathological score caused by DSS ( Figure 3B). These data indicated that TAX reduced the histological injury in DSS-induced colitis mice.

### TAX ameliorated DSS-induced damage to the epithelial and mucosal barrier

**Figure 4:**
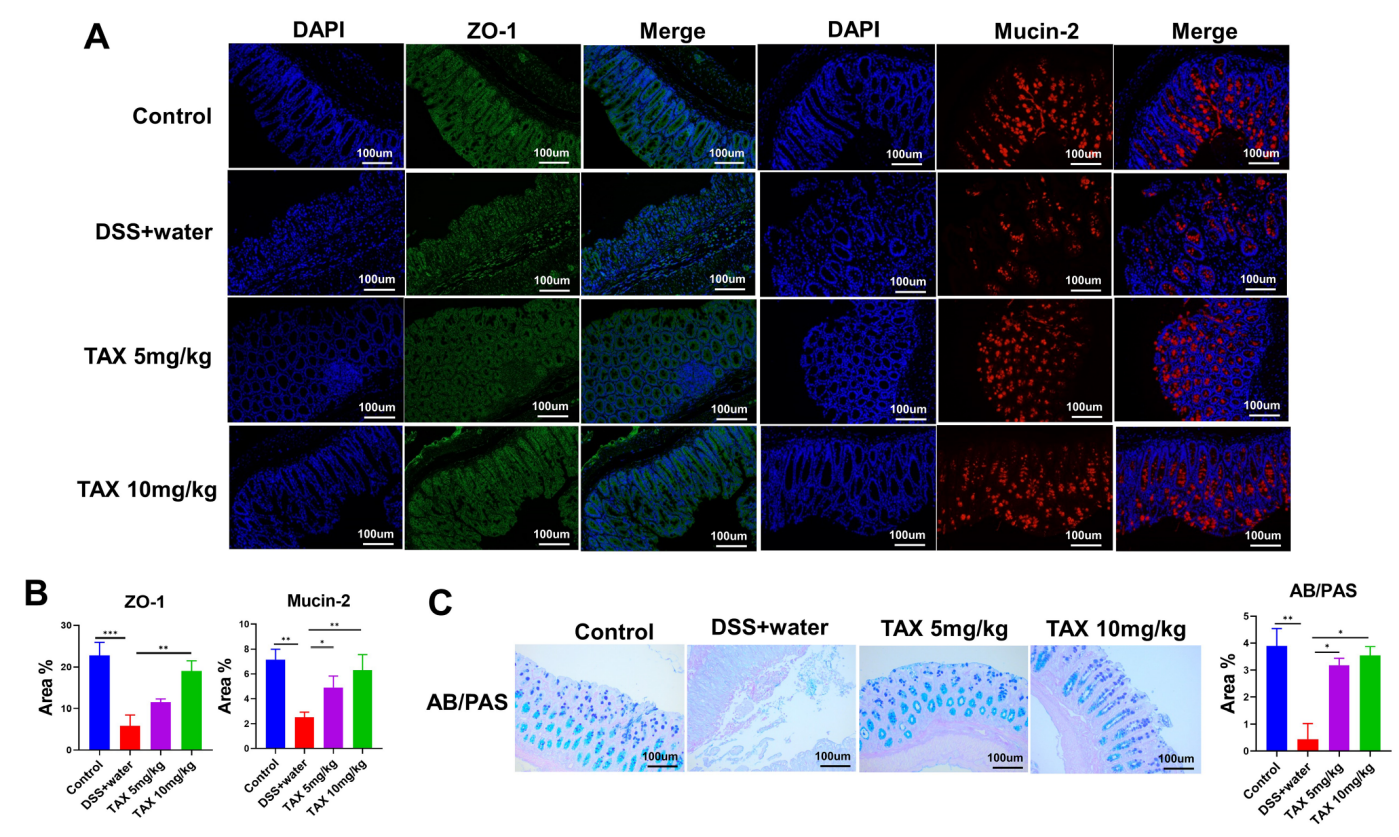
Figure4 TAX exerted beneficial effects on intestinal barrier function in DSS-induced UC mice (A) Immunofluorescence microscopy was used to detect the immunoreactivity of ZO-1 and mucin-2. (B) Analysis of the expression levels of ZO-1 and Mucin-2. (C) AB/PAS staining was used to detect the mucin-producing cells. Data are expressed as the mean±SD. *P<0.05, **P<0.01, and ***P<0.001.

The expressions of the epithelial tight junction protein ZO-1 and mucin protein (Mucin-2) were detected by immunofluorescence microscopy. The number of mucin-producing cells was detected by AB/PAS staining. We found that the immunoreactivities of ZO-1 and Mucin-2 in the colon tissues were significantly inhibited by DSS. However, this was reversed by oral administration of TAX ( Figure 4A,B). Moreover, the number of mucin-producing cells in the DSS+water group was significantly lower than that in the control group, and this was increased after TAX treatment ( Figure 4C). These findings indicated that TAX enhanced the epithelial and mucosal barrier function of DSS-induced colitis.

### TAX altered the similarity and diversity of gut microbiota in DSS-induced colitis

**Figure 5:**
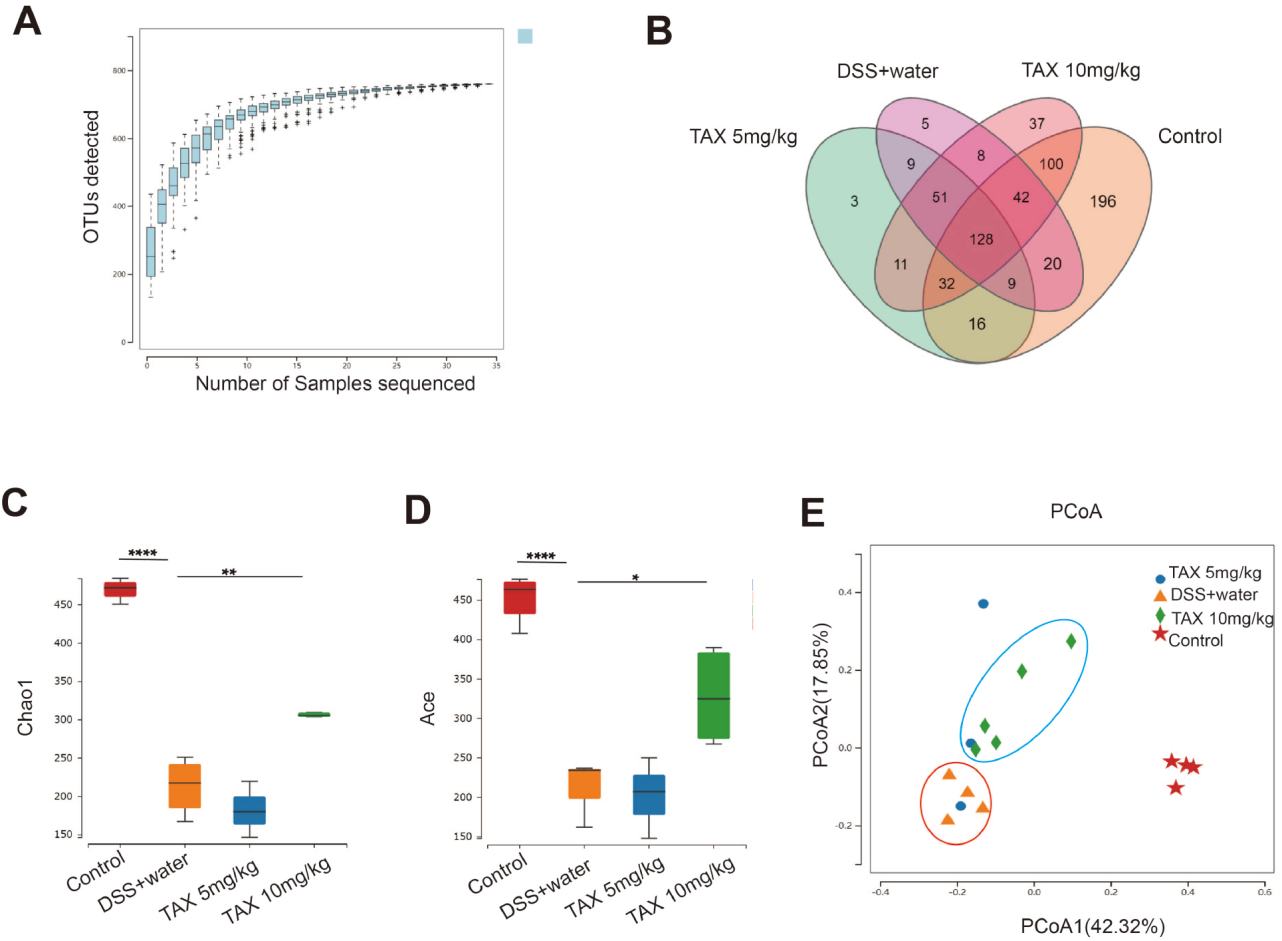
Figure5 TAX altered the similarity and diversity of gut microbiota in DSS-induced colitis (A) OTU rank curve. (B) Venn diagram of OTUs. (C,D) Alpha diversity was estimated by the Chao1 (C) and Ace indexes (D). (E) Principal coordinates analysis (PCoA) plot. Data are expressed as the mean±SD. *P<0.05, **P<0.01, and ****P<0.0001.

The 16S rDNA sequencing was used to evaluate the fecal microbial community, and the similarity of OTUs was >97%. As shown in Figure 5A, the end of the OTU curve tends to be flat, indicating that the sampling volume is sufficient. The Venn diagram result showed that 128 universal OTUs were detected out of 667 total OTUs in all samples. There were 196, 5, 37, and 3 unique OTUs in the control, DSS+water, TAX 10 mg/kg and TAX 5 mg/kg group, respectively ( Figure 5B). Alpha diversity of microbial communities was expressed by Chao1 and Ace indexes. Both Chao1 ( Figure 5C) and Ace indexes ( Figure 5D) were significantly reduced by DSS, but this was counteracted by treatment with 10 mg/kg TAX. The structural shifts of gut microbiota were analyzed by principal coordinate analysis (PCoA) based on uniFrac distance, and the results showed that the microbiota was separated by treatment with DSS and 10 mg/kg TAX ( Figure 5E). These results suggested that TAX altered the similarity and diversity of gut microbiota in DSS-induced colitis.

### TAX changed the composition of gut microbiota in mice with DSS-induced colitis

**Figure 6:**
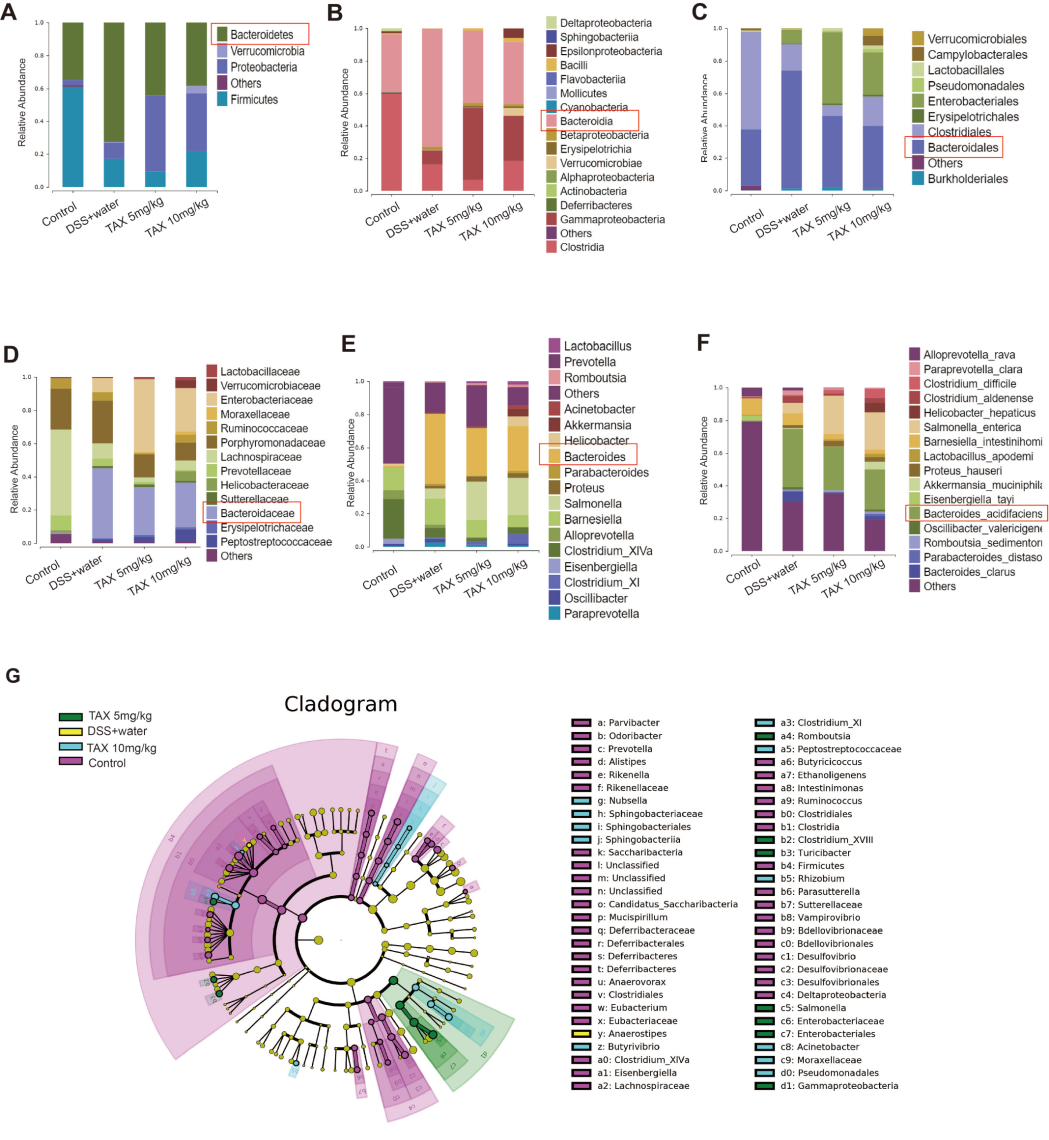
Figure6 TAX changed the composition of gut microbiota in mice with DSS-induced colitis Relative abundance of predominant bacteria was shown at the phylum (A), class (B), order (C), family (D), genus (E), and species (F) levels. (G) Taxonomic cladogram of LEfSe analysis. Different colors indicate the enrichment of the biomarker taxa of the corresponding group. The circle from inside to outside represents the classification level from the phylum to the genus, and the size of the circle indicates the taxa abundance in the community.

The results of gut microbial structure showed that *Bacteroidetes* and *Firmicutes* were the main phyla in the control group at the phylum level. *Bacteroidetes* and *Bacteroidetes*/ *Firmicutes* ratio were significantly increased by DSS, while the alterations were considerably alleviated by TAX supplementation ( Figure 6A). Correspondingly, *Becteroidia* at the class level ( Figure 6B), *Bacteroidetes* at the order level ( Figure 6C), *Bacteroidaceae* at the family level ( Figure 6D), *Bacteroides* at the genus level ( Figure 6E), and *Bacteroides_acidifaciens* at the species level ( Figure 6F) were significantly augmented by the DSS administration, while these alterations were restored by the TAX supplementation. LEfSe taxonomic cladogram ( Figure 6G) was used to identify the microbial communities that play important roles in different groups. At the phylum level, the results reflected the significant roles of the communities of *Firmicutes*, *Deltaproteobacteria*, *Deferribacteres*, and *Candidatus_Saccharibacteria* in the control group ( *P*<0.05). At the genus level, there were significant effects on *Clostridium_XIVa*, *Eisenbergiella*, *Butyricicoccus*, *Ethanoligenens*, *Intestinimonas*, *Ruminococcus*, *Mucispirillum*, *Saccharibacteria*, *Prevotella*, *Alistipes*, *Rikenella*, *Odoribacter*, *Parvibacter*, *Desulfovibrio*, *Parasutterella*, and *Vampirovibrio* in the control group; *Anaerostipes* in the DSS+water group; *Clostridium_XI*, *Nubsella*, and *Acinetobacter* in the 10 mg/kg TAX group; and *Salmonella*, *Clostridium_X VIII*, *Romboutsia*, and *Turicibacter* in the 5 mg/kg TAX group ( *P*<0.05). In addition, LDA was used to determine the significantly different species ( Supplementary Figure S1). The higher the LDA score, the higher the relative abundance of the corresponding group. These results suggested that TAX altered the composition of the colon microbiota in colitis mice.

### Effect of TAX on the function of gut microbiota in mice with DSS-induced colitis

**Figure 7:**
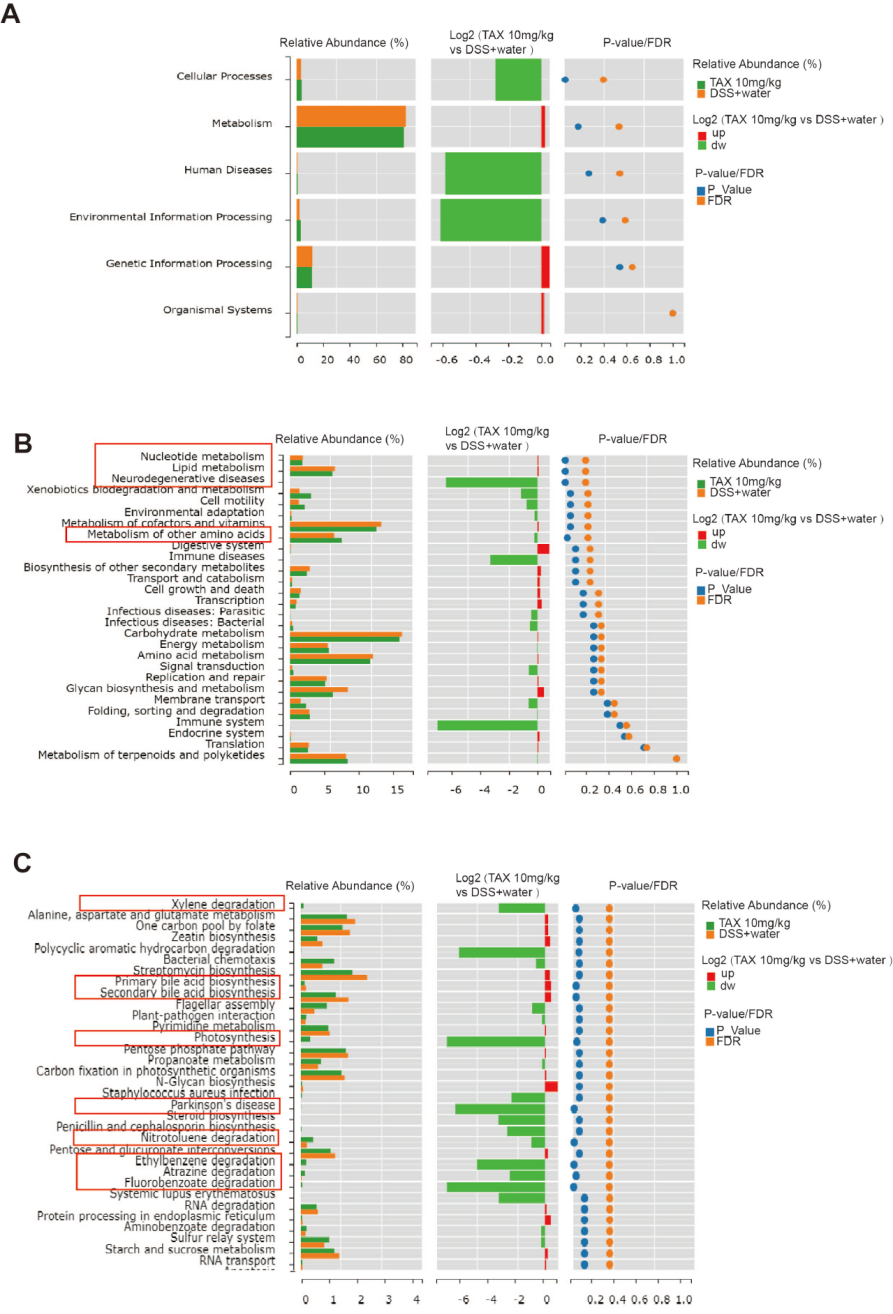
Figure7 The functions of different abundances of microflora Comparisons of the functions of different abundances of microflora between the DSS+water group and 10 mg/kg TAX group at different KEGG levels. (A) Level 1, (B) level 2, (C) level 3.

The function of the microbial community was further predicted by the PICRUSt2 software. The results showed that, in the four groups, the function in the microbial community at KEGG level 1 is mainly focused on metabolism, cellular processes, genetic information processing, human diseases, organismal systems, and environmental information processing ( Supplementary Figure S2A). There was no significant difference in the microbial function at KEGG level 1 between the DSS+water group and 10 mg/kg TAX group ( Figure 7A). However, at KEGG level 2, the nucleotide metabolism, lipid metabolism, neurodegenerative diseases, and metabolism of other amino acids in the DSS group were significantly altered by treatment with 10 mg/kg TAX ( Figure 7B). At KEGG level 3, the functions of microbial communities, including xylene degradation, primary bile acid biosynthesis, secondary bile acid biosynthesis, photosynthesis, Parkinson’s disease, atrazine degradation, fluorobenzoate degradation, ethylbenzene degradation, and nitrotoluene degradation, were significantly changed by the high dose of TAX compared with the DSS group ( Figure 7C). The functions of the four groups of microbial communities at KEGG level 2, and 3 are shown in Supplementary Figure S2B,C. These data indicated that TAX altered the function of gut microbiota in mice with DSS-induced colitis.

## Discussion

Mouse colitis induced by oral administration of DSS resembles some features of human IBD in weight loss, bloody diarrhea, ulcer formation, epithelial cell loss, and neutrophil infiltration [20]. It is a commonly used model for the study of pathogenesis and therapeutic drugs of IBD. In this work, we confirmed that TAX improved the symptoms and pathological features of DSS-induced colitis, exerted beneficial effects on intestinal barrier function, and modulated the composition, diversity, and function of gut microbiota.

Intestinal barrier plays a crucial role in resisting pathogens and toxic substances and maintaining intestinal homeostasis. Intestinal barrier dysfunction is considered to be an important factor for intestinal inflammatory imbalance [21]. As a part of intestinal epithelial barrier, tight junctions are composed of multiple proteins, such as ZO-1. In addition, through the production of mucus, goblet cells are also essential for maintaining the intestinal barrier [21]. Here, TAX was found to increase the number of mucin-producing cells and up-regulate the expressions of ZO-1 and mucin-2 in colon, indicating that TAX exerts beneficial effects on intestinal barrier function in DSS-induced colitis.

Gut microbiota is a complex and abundant ecosystem that mainly colonizes in the gastrointestinal tract, especially in the colon [22]. Numerous studies have been carried to investigate the relationship between gut microbiota and intestinal barrier function [ 23– 25]. The balance of the gut microbiota is essential for maintaining the homeostasis of the intestinal barrier. The gut microbiota at the outer mucus layer regulates the production, secretion, and stratification of intestinal mucus to maintain the integrity of the mucus barrier. Correspondingly, dysbacteriosis leads to impairment of the mucus barrier. In addition, gut microbiota-generated short-chain fatty acids (SCFAs) undergo β-oxidation in colon cells to produce carbon dioxide (CO _2_), which is converted to bicarbonate (HCO _3_^–^) by carbonic anhydrase. This in turn promotes the stratification of the mucus layer [23]. At the same time, the mucus barrier provides a symbiotic environment for gut microbiota [26]. In this study, TAX was found to modulate the gut microbiota of DSS-induced UC mice. We inferred that the improvement of intestinal barrier function might be related to the regulation of gut microbiota by TAX, which needs to be explored in future studies.

Accumulating evidence has demonstrated the essential role of gut microbiota in the development and evolution of intestinal inflammation [ 22, 27, 28]. A series of data from animal models and clinical studies have confirmed the dysbiosis of gut microbiota in UC [ 22, 29], which is manifested by decreased diversity, irregular composition, and changes in spatial distribution and/or function of the gut microbiota [22]. In particular, the reduced diversity of fecal microbiota is the most consistent in UC [ 30– 32]. In addition, compared with healthy individuals, patients with UC showed fewer *Firmicutes* [33] and a higher abundance of *Bacteroidetes* [34] at the level of phylum. Studies about the alteration of gut microbiota at the genus or species level in UC have been extensively carried out. At the genus or species level, UC patients showed reduced amounts of bacterial groups from the *Clostridium cluster XIV* as compared with the healthy controls [34]. In UC models, the relative abundance of *Bacteroides*, *Corynebacterium*, *Proteus*, *Helicobacter*, *Staphylococcus*, and *Streptococcus* was recently found to be increased [ 35, 36]. In this study, we found that the diversity of microbial communities was restrained by DSS administration, while this was reversed by TAX treatment. In addition, TAX also shifted the microbiota composition. The abundance of *Bacteroidetes* and the *Bacteroidetes*/ *Firmicutes* ratio were significantly increased by DSS, but decreased by TAX treatment. This is consistent with a previous report, in which taxifolin was found to inhibit the growth of *Bacteroides*, thereby reducing intestinal inflammation [37]. This indicated that TAX modified the diversity and composition of gut microbiota and improved symptoms of colitis.

Alterations in the gut microbiota are usually related to a dynamic change in the overall metabolome. SCFAs (mainly acetate, propionate, and butyrate) are important metabolites of gut microbes. They are the main source of energy for colonocytes and intestinal immune cells, and play a beneficial role in anti-inflammatory reaction, increasing the integrity of epithelial cells and maintaining intestinal homeostasis [ 38– 40]. Acetate and propionate are mostly produced by members of *Bacteroidetes*, while *Firmicutes* mainly produce butyrate in the human intestine [ 41, 42]. Primary bile acids synthesized from cholesterol are converted into secondary bile acids by bacteria in the intestine. Bile acids and their receptors mediate the communications of intestinal microbiota with the intestinal immune system [43] and regulate immunity by downregulating the expressions of pro-inflammatory cytokines in monocytes, macrophages, dendritic cells, and liver macrophages [44]. In addition, the gut microbiota and their metabolites also affect the host’s innate and adaptive immunity [ 22, 45, 46]. Our functional analysis of gut microbiota showed that metabolism is the primary function of the microbial community at KEGG level 1, and the results of the KEGG level 2 analysis also indicated the differential function was mainly associated with metabolism. Previous reports have shown that *Firmicutes* and *Bacteroidetes* may be involved in the metabolism of host lipid and bile acid, and a decrease in the *Bacteroidetes*/ *Firmicutes* ratio is associated with disorders of lipid and bile acid metabolism [ 47– 49]. In the present study, we found that the abundance of *Firmicutes* and *Bacteroidetes* and the ratio of *Bacteroides*/ *Firmicutes* in the DSS+water group were changed, and lipid and bile acid metabolism were also affected. These changes are consistent with the above research reports. Importantly, TAX intervention reduced the abundance of *Bacteroidetes* and the *Bacteroides*/ *Firmicutes* ratio, and modulated the function of lipid and bile acid metabolism. This indicates that metabolism function is a potential target for TAX to manage intestinal inflammation and colitis. In addition, we also found that there were other functions in the 10 mg/kg TAX group and the DSS+water group, such as functions related to nucleotide metabolism, neurodegenerative diseases, and degradation of some compounds, which were also significantly different. However, in this study we did not find any functional differences regarding SCFAs. It is worth noting that 10 mg/kg TAX exhibited better effects than 5 mg/kg TAX in ameliorating UC symptoms, improving intestinal barrier function, and regulating the gut microbiota, which indicated that the effect of TAX on UC might be dose-dependent. Further studies are needed to clarify whether TAX becomes more effective with increasing doses.

In this study, the PICRUSt2 software was applied to predict the function of the microbial community. Unlike PICRUSt1, PICRUSt2 contains an updated and larger gene family and reference genome database. It is no longer necessary to use the GreenGene-annotated OTU table as input like PICRUSt1. It can directly read the representative sequence of OTU and automatically complete the species annotation, and further predict the community function based on the species abundance composition [50]. PICRUSt2 provides higher accuracy and flexibility for the metagenomic inference of marker genes. However, there are some limitations. For example, PICRUSt2 can only predict genes that are already in the input database. This is also one of the limitations of the study. In the future, genomic prediction needs to be further optimized to improve the accuracy of functional prediction.

In conclusion, our data demonstrated that oral administration of TAX protected against colitis in mice through improving intestinal barrier function and modulating gut microbiota dysbiosis. This study provides new insights into the biological functions and therapeutic potential of TAX in the treatment of UC.

## Supporting information

Supplementary

## Supplementary Data

Supplementary Data is available at *Acta Biochimica et Biphysica Sinica* online.
