# Supplementary material for: Taraxasterol ameliorates dextran sodium sulfate-induced murine colitis via improving intestinal barrier and modulating gut microbiota dysbiosis: Taraxasterol ameliorates colitis via intestinal barrier and gut microbiota dysbiosis
Source: Acta Biochim Biophys Sin (Shanghai). 2022 Mar 3;54(3):340–9. doi: 10.3724/abbs.2022019 (PMC9827818; doi:10.3724/abbs.2022019)
Supplement: Supplementary [file Supplementary.docx]

**
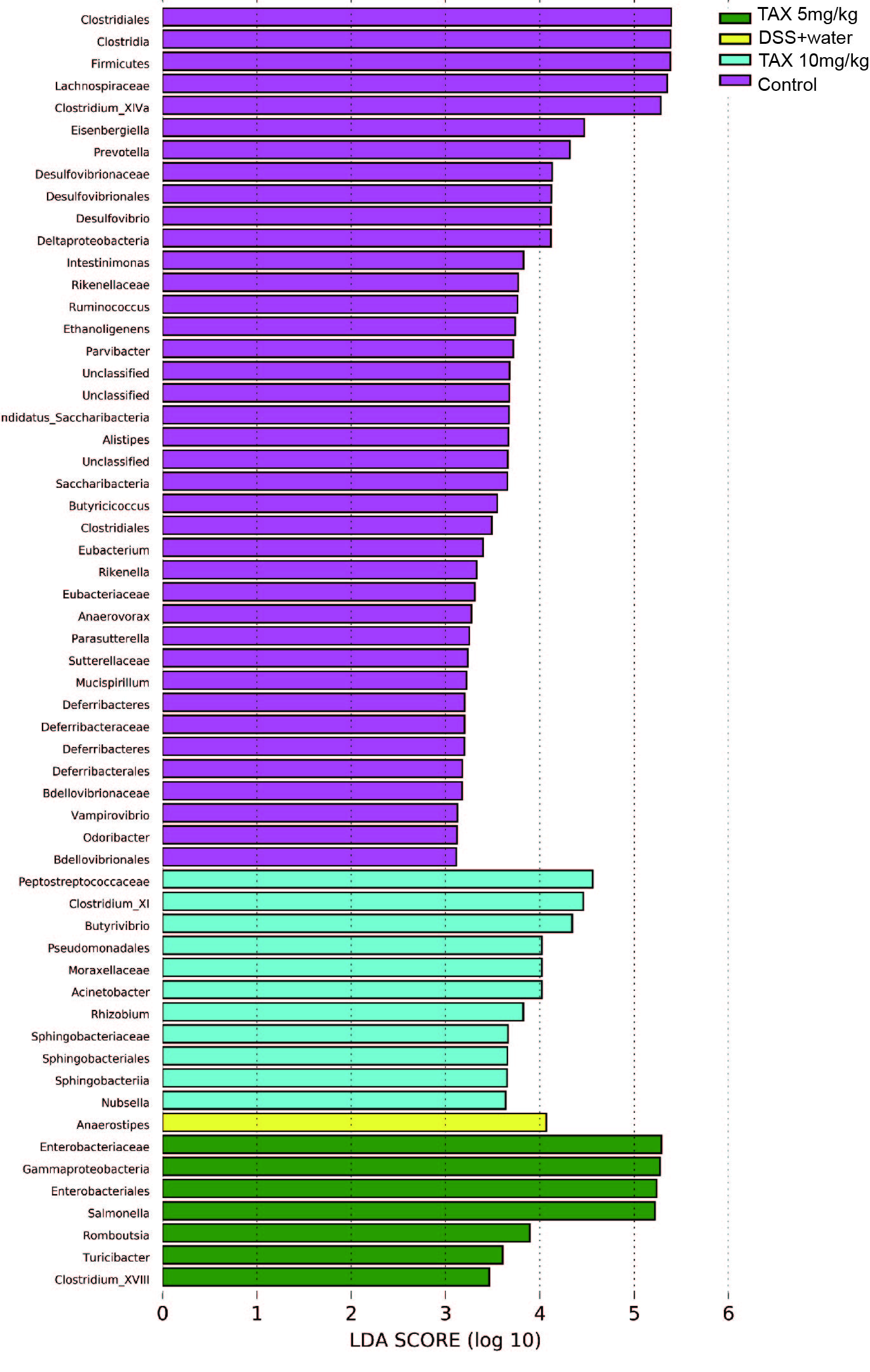
**

**Supplementary Figure S1.** Linear discriminant analysis (LDA) score for different taxa abundances.


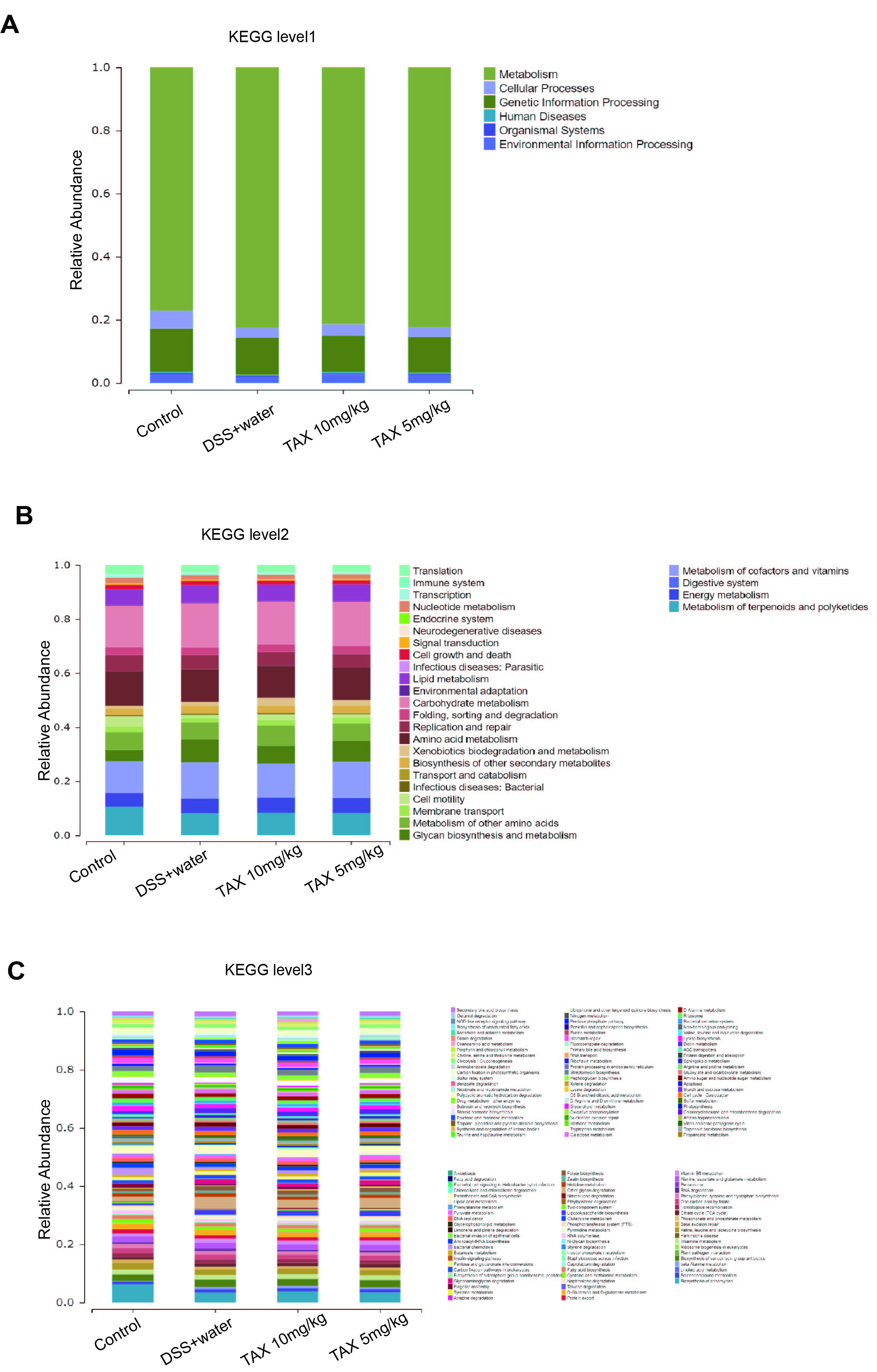


**Supplementary Figure S2.** The different function of microflora in the four groups at KEGG levels 1 **(A)**, 2 **(B),** and 3 **(C)**.
